# Prognosis prediction and tumor immune microenvironment characterization based on tryptophan metabolism-related genes signature in brain glioma

**DOI:** 10.3389/fphar.2022.1061597

**Published:** 2022-11-01

**Authors:** Shuxin Zhang, Siliang Chen, Zhihao Wang, Junhong Li, Yunbo Yuan, Wentao Feng, Wenhao Li, Mina Chen, Yanhui Liu

**Affiliations:** ^1^ Department of Neurosurgery, West China Hospital of Sichuan University, Chengdu, Sichuan, China; ^2^ Department of Head and Neck Surgery, Sichuan Cancer Hospital and Institute, School of Medicine, University of Electronic Science and Technology of China, Chengdu, China; ^3^ Department of Neurosurgery, Chengdu Second People’s Hospital, Chengdu, Sichuan, China; ^4^ State Key Laboratory of Biotherapy, Neuroscience and Metabolism Research, West China Hospital, Sichuan University, Chengdu, China

**Keywords:** tryptophan, glioma, metabolism, prognosis, immune infiltration, immune checkpoint inhibitor, tumor microenvironment

## Abstract

Glioma is the most common malignant tumor in the central nervous system with no significant therapeutic breakthrough in recent years. Most attempts to apply immunotherapy in glioma have failed. Tryptophan and its metabolism can regulate malignant features of cancers and reshape immune microenvironment of tumors. However, the role of tryptophan metabolism in glioma remains unclear. In current study, we explored the relationships between the expression pattern of tryptophan metabolism-related genes (TrMGs) and tumor characteristics, including prognosis and tumor microenvironment of gliomas through analyzing 1,523 patients’ samples from multiple public databases and our own cohort. Based on expression of TrMGs, K-means clustering analysis stratified all glioma patients into two clusters with significantly different TrMG expression patterns, clinicopathological features and immune microenvironment. Furthermore, we constructed a tryptophan metabolism-related genes signature (TrMRS) based on seven essential TrMGs to classify the patients into TrMRS low- and high-risk groups and validated the prognostic value of the TrMRS in multiple cohorts. Higher TrMRS represented for potentially more active tryptophan catabolism, which could subsequently lead to less tryptophan in tumor. The TrMRS high-risk group presented with shorter overall survival, and further analysis confirmed TrMRS as an independent prognostic factor in gliomas. The nomograms uniting TrMRS with other prognostic factors manifested with satisfactory efficacy in predicting the prognosis of glioma patients. Additionally, analyses of tumor immune landscapes demonstrated that higher TrMRS was correlated with more immune cell infiltration and “hot” immunological phenotype. TrMRS was also demonstrated to be positively correlated with the expression of multiple immunotherapy targets, including PD1 and PD-L1. Finally, the TrMRS high-risk group manifested better predicted response to immune checkpoint inhibitors. In conclusion, our study illustrated the relationships between expression pattern of TrMGs and characteristics of gliomas, and presented a novel model based on TrMRS for prognosis prediction in glioma patients. The association between TrMRS and tumor immune microenvironment of gliomas indicated an important role of tryptophan and its metabolism in reshaping immune landscape and the potential ability to guide the application of immunotherapy for gliomas.

## Introduction

Glioma, a type of malignant tumor arising from glial cells, is responsible for approximately 80% of all malignant tumors in the central nervous system (Ostrom et al., 2021). Current standard treatment regime for glioma consists of surgery, radiotherapy, and chemotherapy (Stupp et al., 2005; Weller et al., 2021). However, even with entire process of standard treatment, the prognosis of glioma patients remains unsatisfactory, especially for glioblastoma, which manifests with highly malignant features and only achieved a median overall survival of fewer than 2 years (Chinot et al., 2014; Gilbert et al., 2014; Stupp et al., 2015). Hence, plenty studies devoted to exploring novel therapies to improve prognosis of glioma patients, one of which was immunotherapy. Immunotherapy, aiming to restrict the immune escape phenomenon of tumors and enhance the anti-tumor immunity executed by immune cells, has been proved with ability to improve patients’ overall survival in numerous cancers, including melanoma (Larkin et al., 2015), cervical cancer (Tewari et al., 2022), gastric cancer (Janjigian et al., 2021), and lung cancer (Reck et al., 2016). However, almost all trials of immune checkpoint inhibitors (ICIs) did not endorse improvement of overall survival in glioblastoma patients (Reardon et al., 2020; Lim et al., 2022; Omuro et al., 2022). One of the potential reasons of these failures is immunologically quiescent environment of the central nervous system. But the metastatic brain tumor patients could benefit from ICIs therapy (Tawbi et al., 2018; Hendriks et al., 2019), suggesting that ICIs could deliver robust anti-tumor effects into CNS and the distinctive immune landscape of gliomas may be a potential reason for failures of ICIs. Besides, the application of neoadjuvant ICIs in glioblastoma was proved could enhance the immune response and reshape the immune landscape (Cloughesy et al., 2019; Schalper et al., 2019). Furthermore, multiple factors, including lifestyle, metabolic disorders, and social behaviors, could also influence the expression of checkpoint inhibitors and responses to immunotherapy (Deshpande et al., 2020). For example, obesity could upregulate the production of leptin and consequently promoted PD-1 expression on T-cells, leading to high response rate to immunotherapy in overweighted individuals (Wang et al., 2019). Besides, the responses to immunotherapy were observed more effective in smokers compare to never smoking individuals in lung cancer, which might result from high mutation rate in smokers (Abdel-Rahman, 2018). Hence, exploring possible pathways to reshape the immune landscape and enhance the response to immunotherapy can contribute to reinforcing the effects of immunotherapy and improving prognosis of glioma patients.

Tryptophan, an essential amino acid for human, is gained exclusively from diary intake. Tryptophan, together with its metabolites, is proved to play critical roles in multiple physiological processes, including cell maintenance and growth (Platten et al., 2019). Furthermore, tryptophan and its metabolites could also function as neurotransmitter and signalling molecules (Cervenka et al., 2017). Furthermore, shortage of tryptophan would activate the General control nonderepressible 2 (GCN2) pathway and lead to dysfunction of antigen-presenting cells and T-cells (Munn et al., 2005). Reduced tryptophan level was detected in multiple cancers (Huang et al., 2002; Schroecksnadel et al., 2005; Weinlich et al., 2007; Suzuki et al., 2010), suggesting potential role of tryptophan in cancers. In the tryptophan metabolism, over 95% of free tryptophan is degraded by the kynurenine pathway (Le Floc’h et al., 2011; van der Goot and Nollen, 2013). Indoleamine-2,3-dioxygenase (IDO) and tryptophan-2,3-dioxygenase (TDO), which catalyzes the same reaction, are rate-limiting enzymes in the kynurenine pathway (Grohmann et al., 2017). IDO was confirmed with immunosuppressive effect (Munn et al., 1998), and silencing the expression of IDO could enhance the antitumor immunity (Yen et al., 2009). TDO was also proved with similar immunosuppressive effects by inhibiting proliferation of T-cells and blocking infiltration of immune cells (Opitz et al., 2011; Pilotte et al., 2012). These studies revealed that tryptophan plays a critical role in tumor progression and process of antitumor immunity, and the metabolism of tryptophan has a significant impact on immunological feature of tumors. However, the role of tryptophan metabolism in the progression and immune landscape of glioma was not well elucidated.

In our current study, we utilized multiple glioma patients’ cohort, including TCGA, CGGA, REMBRANDT, and our own cohort, to explore relationship between the expression pattern of tryptophan metabolism-related genes (TrMGs) and the characteristics of gliomas. Besides, we constructed a tryptophan metabolism-related gene signature (TrMRS) to assess the clinical significance of TrMG expression profile. Furthermore, we performed multiple analyses to elucidated relationship between the expression of tryptophan metabolism-related genes and the landscape of tumor immune microenvironment of gliomas. Based on these analyses, we look forward to exploring the potential applications of tryptophan metabolism in improving responses to immune checkpoint inhibitors and guiding selection of immunotherapy in glioma patients.

## Materials and methods

### RNA-sequencing and clinicopathological data collection and preprocessing

The RNA-sequencing and clinicopathological data enrolled in this study were acquired from three public databases and an own cohort. Patients with primary gliomas included in this study. Here, the notion of gliomas is restricted to astrocytomas, oligodendrogliomas, and glioblastomas. Those with recurrent gliomas or age < 18 were excluded from this study. In total, 662 primary gliomas from the Cancer Genome Atlas (TCGA, of which 655 had survival data) were included in our study, and the fragments per kilobase million (FPKM) and survival data of them were downloaded from the TCGA website (https://portal.gdc.cancer.gov/). Another 415 primary gliomas were from the Chinese Glioma Genome Atlas (CGGA) 693 cohort, and the REMBRANDT cohort which consists of 369 primary gliomas. FPKM data of the CGGA cohort and array data of REMBRANDT cohort were obtained from the CGGA website (http://www.cgga.org.cn/). For data preprocessing, the genes with too low expression levels (FPKM maximum < 0.1 or standard deviation < 0.01) were excluded from subsequent analyses.

Our own cohort included 77 primary glioma patients from West China Hospital (WCH). We obtained their tumor tissues during craniotomy and sequenced mRNA of these tumor tissues. Subsequently, we used STAR to quantify the mRNA sequencing data and normalized them to FPKM. Survival data of these patients were acquired by regular follow-up, and the overall survival (OS) was defined as the period from surgery to death or the time of last follow-up (censored value). Additionally, the patients younger than 18 years old were excluded from analyses in all four cohorts. The detailed clinicopathological information of these patients is given in Table 1.

**TABLE 1:** Clinicopathological characteristics of patients in TCGA, CGGA, REMBRANDT, and WCH cohort.

Characteristics	TCGA (N = 662)	CGGA (N = 415)	REMBRANDT (N = 369)	WCH (N = 77)
Age: mean (range)	46 (18–89)	43 (19–76)	52 (22–87)	46 (19–77)
Gender				
Female	282 (42.6%)	176 (42.4%)	118 (32.0%)	30 (39.0%)
Male	380 (57.4%)	239 (57.6%)	196 (53.1%)	47 (77.0%)
NA	0	0	55 (14.9%)	0
Histology				
Astrocytoma	341 (51.5%)	182 (43.9%)	133 (36.0%)	22 (28.6%)
Oligodendroglioma	167 (25.2%)	94 (22.7%)	59 (16.0%)	21 (27.3%)
Glioblastoma	154 (23.3%)	139 (33.5%)	177 (48.0%)	34 (44.2%)
Grade				
G2	214 (32.3%)	134 (32.3%)	88 (23.8%)	29 (37.7%)
G3	237 (35.8%)	142 (34.2%)	66 (17.9%)	14 (18.2%)
G4	154 (23.3%)	139 (33.5%)	177 (48.0%)	34 (44.2%)
NA	57 (8.6%)	0	38 (10.3%)	0
IDH status				
WT	236 (35.6%)	169 (40.7%)	NA	42 (54.5%)
Mutant	421 (63.6%)	207 (49.9%)	NA	35 (45.5%)
NA	5 (0.8%)	39 (9.4%)	NA	0
1p/19q codeletion				
Non-codel	488 (73.7%)	267 (64.3%)	148 (40.1%)	43 (55.8%)
Codel	167 (25.2%)	88 (21.2%)	24 (6.5%)	19 (24.7%)
NA	7 (1.1%)	60 (14.5%)	197 (53.4%)	15 (19.5%)
TERT promoter status				
Mutant	340 (51.4%)	NA	NA	23 (29.9%)
WT	156 (23.6%)	NA	NA	30 (39.0%)
NA	166 (25.1%)	NA	NA	24 (31.2%)
MGMT promoter status				
Unmethylated	157 (23.7%)	141 (34.0%)	NA	13 (16.9%)
Methylated	472 (71.3%)	195 (47.0%)	NA	35 (45.5%)
NA	33 (5.0%)	79 (19.0%)	NA	29 (37.7%)
ATRX status				
Mutant	192 (29.0%)	NA	NA	53 (68.8%)
WT	459 (69.3%)	NA	NA	22 (28.6%)
NA	11 (1.7%)	NA	NA	2 (2.6%)

TCGA, the cancer genome atlas; CGGA, chinese glioma genome atlas; WCH, west china hospital; IDH, isocitrate dehydrogenase; TERT, telomerase reverse transcriptase; MGMT, O6-methylguanine-DNA, methyltransferase; ATRX, alpha-thalassemia x-linked intellectual disability syndrome; WT, wild type; NA, not available.

### K-means clustering analysis based on expression pattern of tryptophan metabolism-related genes

By searching the Molecular Signature Database (MSigDB) with the keyword “tryptophan metabolism” or “tryptophan metabolic process,” we identified 56 tryptophan metabolism-related genes (TrMGs), and 44 of which were kept after excluding the genes with low expression level. Subsequently, we performed unsupervised K-means clustering analysis to illuminate the distinctive tryptophan metabolism patterns in gliomas based on the expression patterns of tryptophan metabolism-related genes. We utilized the R package “factoextra” to determine the optimal number of clusters, which corresponded to the maximum average silhouette width (average distances of points to the centroids of clusters that it does not belong to minus the distance of points to the centroid of the cluster that it belongs to). To visualize the different expression patterns of TrMGs in each cluster, we conducted the t-Distributed Stochastic Neighbor Embedding (tSNE) analysis. Additionally, we utilized the TrMGs expression and cluster labels based on the TCGA cohort to construct a naïve Bayes classifier, and then stratified the patients of the other cohort into different clusters using this classifer.

### Construction and validation of the risk signature based on tryptophan metabolism-related genes

Based on the expression of TrMGs, we constructed a risk signature system to elucidate the relationship between TrMGs and gliomas. First, we split the patients of TCGA cohort into training and validation tests with a ratio of 6:4. All the other three cohorts were utilized as validation sets. In the training set, we selected the TrMGs using the Least Absolute Shrinkage And Selection Operator (LASSO) Cox regression analysis. The TrMGs were determined as essential TrMGs in glioma if their coefficient was not zero at the lambdas corresponding to maximum C-index in over 80 random repetitions of LASSO Cox regression out of 100. Furthermore, a final multivariate Cox regression model was fitted to the training set with essential TrMGs. The tryptophan metabolism-related genes risk signature (TrMRS) was calculated using the following formula:

$$TrMRS\;Risk\;Signature=\underset{i=1}{\sum }\left({\beta_{i}*\mathrm{Exp}}_{i}\right)$$

In this calculating formula, *β* stand for the coefficient of each essential TrMG as fitted by the final multivariate Cox regression model, and Exp represented for the expression level of each essential TrMG. Subsequently, we determined the optimal TrMRS cut-off value by “surv_cutpoint” in the R package “survminer” with group proportion ≥ 0.3 for each dataset. Based on the optimal cut-off value, we allocated all patients into TrMRS low-risk or high-risk group. Additionally, we depicted the receiver operating characteristic (ROC) curve in validation sets of 1, 2, and 3-years survival and computed the area under the ROC curve (AUC) using the R package “timeROC.”

### Analyses of gene alternations and copy number variation

For the analyses of gene alternations and copy number variations (CNVs), we obtain the data of gene alterations and CNVs from the cBioPortal database (https://www.cbioportal.org/) for the TCGA cohort. The R package “maftools” was utilized to illustrate the different patterns of gene alterations and tumor mutation burdens (TMB) between different K-means clusters and TrMRS risk groups. Furthermore, the Genomic Identification of Significant Targets in Cancer (GISTIC) score was used to assess the different CNV levels in different clusters and risk groups.

### Gene set enrichment analysis and comprehensive analysis of tumor immune microenvironment landscape

To interpret the biological functions of the differential transcriptomes between clusters and TrMRS risk groups, we utilized the over-representation and gene set enrichment analysis (GSEA) to evaluate the differentially expressed genes (DEGs) with the R package “clusterProfiler”. R package “limma” was used to identify the DEGs between different clusters and risk groups. In the process of DEGs identification for GSEA, we stratified the patients into TrMRS high- and low-risk groups based on cut-off values of each cohort. Those genes with adjusted *p*-value < 0.05 and |log_2_FC| > 0.5 were identified as DEGs. Furthermore, we used the R package “GSVA” to convert the logFPKM matrix of genes to pathway expression matrix. We identified the differentially expressed pathways between different clusters and risk groups with the “limma” package. To calculate the infiltration fraction of immune cells in glioma, we utilized the CIBERSORTx (https://cibersortx.stanford.edu/) and TIMER 2.0 [http://timer.comp-genomics.org/, which is based on signature genes correlated with estimated tumor purity and immune cell fractions (Li et al., 2016)]. Additionally, the Estimation of Stromal and Immune Cells in Malignant Tumor tissues using Expression data (ESTIMATE) was used to evaluate the infiltration of stromal and immune cells in tumor microenvironment and calculate the stromal, immune and ESTIMATE scores (Yoshihara et al., 2013). In this algorithm, the stromal-related genes were selected from the non-hematopoiesis-related genes that were differentially expressed between tumor cell fraction and match stromal cells fraction separated by laser capture microdissection in multiple cancers. Furthermore, the tumor purity data published by Aran et al. (2015) which included ESTIMATE score-based tumor purity and consensus purity estimation (CPE), were used to represent the tumor purity in gliomas. Another previously published algorithm was utilized to compute the tumor immunological phenotype (TIP) gene signature (Wang et al., 2021). Based on TIP signature, we identified immunological phenotypes of gliomas and distinguish relatively “hot” tumors from “cold” tumors. Furthermore, we also utilized the Tumor Immune Dysfunction and Exclusion (TIDE) suite (http://tide.dfci.harvard.edu/) to predict response to immune checkpoint inhibitors therapy in gliomas.

### Construction of nomogram based on tryptophan metabolism-related genes and other prognostic factors

To determine prognostic factors in glioma, we used univariate and multivariate Cox regression analyses. We firstly enrolled TrMRS and other potential prognostic factors, including tumor grade, age, chemotherapy, radiotherapy, KPS, gender, 1p/19q codeletion, and IDH mutation, into the univariate Cox regression analysis. Subsequently, those factors with a *p*-value < 0.05 in univariate analysis were enrolled into multivariate Cox regression analysis to identify independent prognostic factors. Those factors with a *p*-value < 0.1 in multivariate Cox regression analysis were identified as independent factors and enrolled into construction of nomograms. The R package “rms” was utilized to construct the nomograms. The calibration curves were used to evaluate the efficacy of nomograms for prognosis prediction in glioma patients.

### Statistical Analysis

In all proceedings of bioinformatic analyses, we used the R software (version 4.2.1). To assess the differences between clusters or risk groups for continuous variables, we used the Wilcoxon rank sum test. To assess the differences for categorical variables, we used the chi-square test. The R package “survminer” was used to deliver survival analysis and generate Kaplan-Meier (K-M) curves, which were tested for differences by log-rank test. The “coxph” function in the R package “survival” was applied to conduct Cox regression analyses. The R package “glmnet” was utilized to perform the LASSO Cox regression analysis. T Iterative Grubbs tests were applied to expel the outliers in liner regression analysis.

### Ethical approval, consent to participate, and data availability

Tumor samples and clinical data collection and use were performed strictly with ethics regulations and approved by the institutional review board of West China Hospital (No. 2018.569) based on local ethics regulations and the 1964 Helsinki declaration and its later amendments. In addition, the patients signed written consent for tumor tissue collection and processing. The sequencing data of West China Hospital generated in this study are available at the Genome Sequence Archive for Humans: accession code HRA002839 (access link: https://ngdc.cncb.ac.cn/gsa-human/s/JQssVoV1).

## Results

### K-means clustering analysis based on tryptophan metabolism-related genes

**FIGURE 1:**
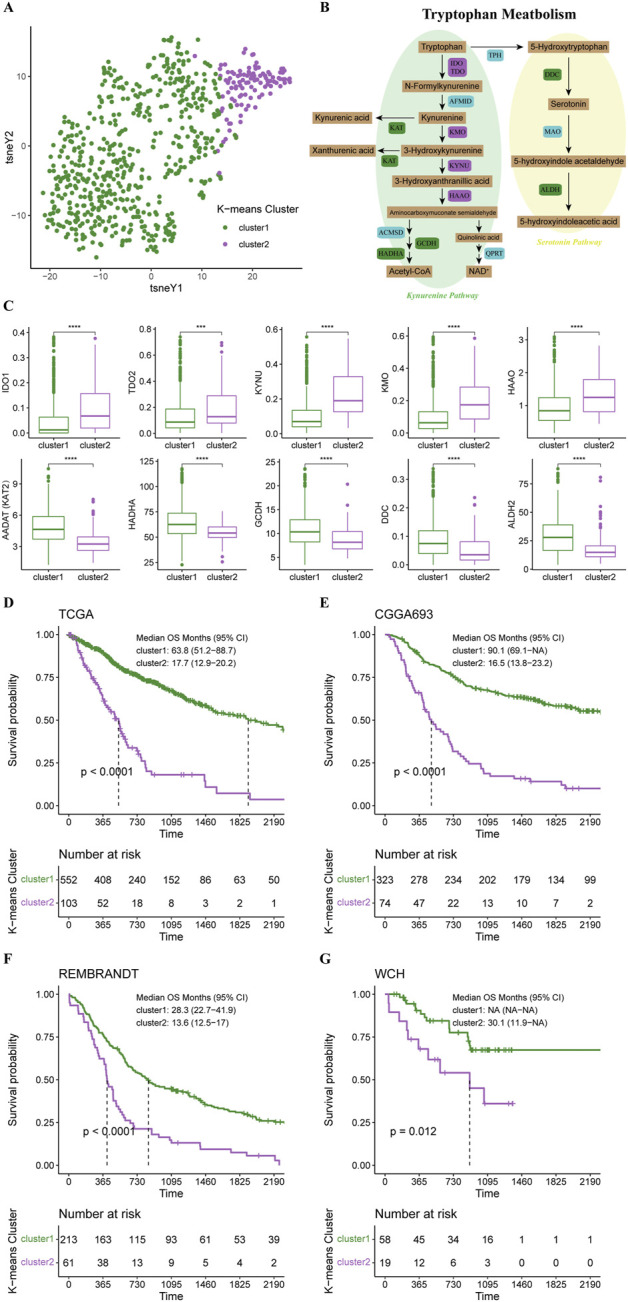
K-means clustering of gliomas based on expression of TrMGs. **(A)** TrMGs expression t-SNE. **(B)** Schematic diagram for tryptophan metabolism **(C)** The expression levels of ten important TrMGs between two K-means clusters (cluster 1: *N* = 556, cluster 2: *N* = 106). **(D)** Kaplan-Meier Curve based on K-means clusters in TCGA, **(E)** CGGA, **(F)** REMBRANDT, and **(G)** WCH cohorts. IDO, indoleamine 2,3-dioxygenase; TDO, tryptophan 2,3-dioxygenase; AFMID, arylformamidase; KMO, kynurenine 3-monooxygenase; KYNU, kynureninase; KAT, kynurenine aminotransferase; HAAO, 3-hydroxyanthranilic acid oxygenase; ACMSD, aminocarboxymuconate semialdehyde decarboxylase; GCDH, glutaryl-CoA dehydrogenase; HADHA, hydroxyacyl-CoA dehydrogenase trifunctional multienzyme complex subunit alpha; QPRT, quinolinic acid phosphoribosyltransferase; TPH, tryptophan hydroxylase; DDC, dopa decarboxylase; MAO, monoamine oxidase; ALDH, aldehyde dehydrogenase.

Based on 44 identified tryptophan metabolism-related genes, we conducted an unsupervised K-means clustering analysis in the TCGA cohort. Then the gliomas in the TCGA cohort were stratified into two clusters according to the average silhouette widths as described in the Material and Method section (Supplementary Figure S1A). Distinctions in TrMGs expression profile between two clusters were illustrated by tSNE analysis (Figure 1A). The expression levels of TrMGs in each glioma was given out in heatmap ordered by clusters (Supplementary Figure S1B). The critical metabolites and enzymes in two branches of tryptophan metabolism, kynurenine pathway and serotonin pathway, were interpreted in a schematic diagram (Figure 1B). The differences in the expression levels of ten important TrMGs involved in the tryptophan metabolism were also illustrated (Figure 1C). The cluster 2 was demonstrated with higher expression of IDO1, TDO2, KYNU, KMO, and HAAO, which were critical enzymes in the kynurenine pathway for tryptophan metabolism. Besides, KAT2, HADHA, GCDH, DDC, and ALDH2 were highly expressed in cluster 1, suggesting more consumption of metabolites of kynurenine pathway and more active serotonin pathway. The differences in the expression levels of each TrMG were shown in Supplementary Figure S1C.

Survival analysis revealed that the prognosis of cluster 1 was significantly better than cluster 2 (median OS, 63.8 vs. 17.7 months) (Figure 1D). The patients in the CGGA, REMBRANDT, and WCH cohorts were also stratified into two clusters based on the naïve Bayes clustering classifier train with the TCGA data. Survival analyses in these three cohorts also revealed significantly better prognosis in cluster 1 (Figures 1E–G), suggesting that the TrMG expression patterns were robust among different glioma cohorts.

**FIGURE 2:**
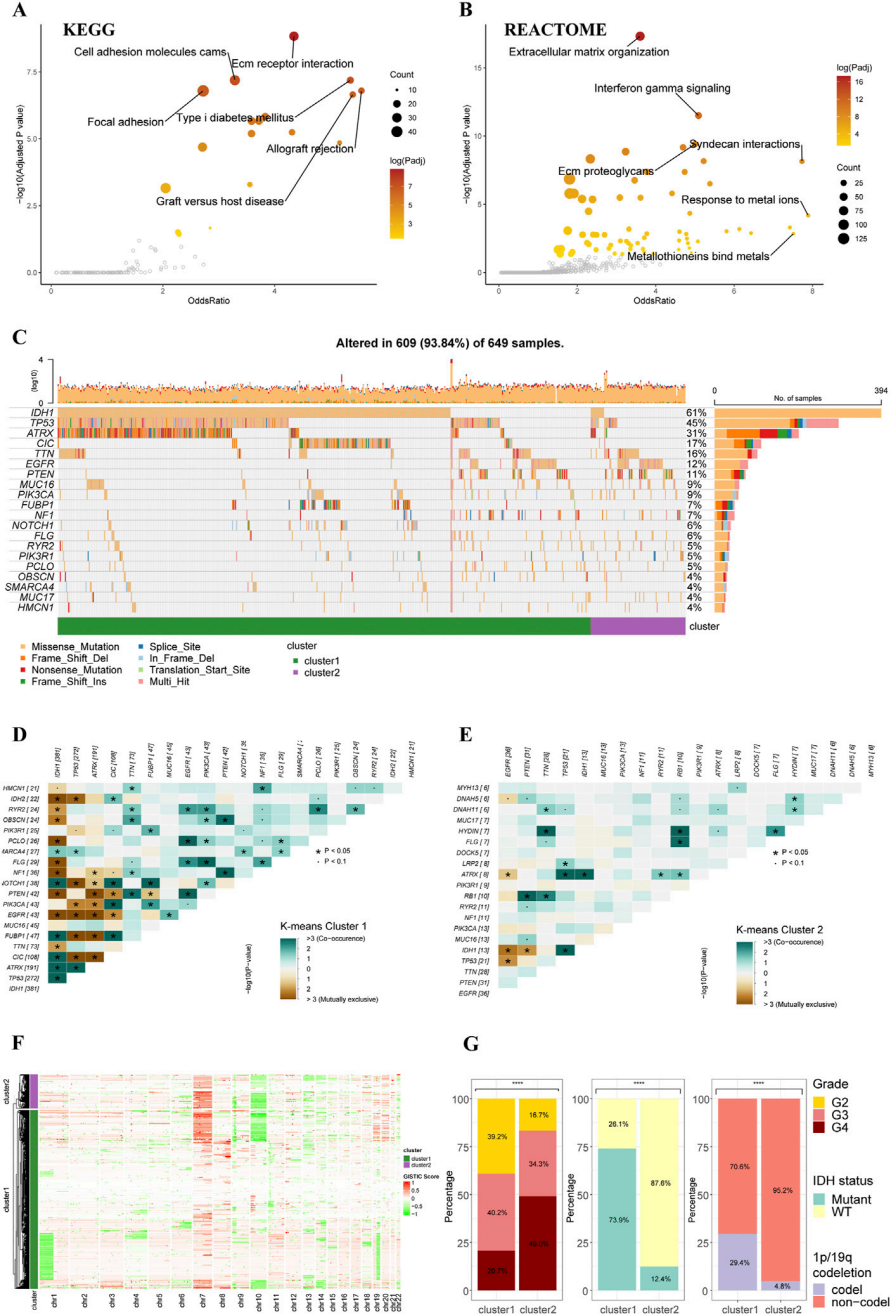
Functional enrichment and clinicopathological features of the K-means clusters. **(A)** Pathways with high odds ratio and confidence in the KEGG gene sets. **(B)** Pathways with high odds ratio and confidence in the REACTOME gene sets. **(C)** Top 20 mutated genes of the K-means clusters. **(D,E)** Co-occurrence and mutually exclusive of the gene mutations in cluster 1 **(D)** and cluster 2 **(E)**. **(F)** Heatmap of copy number variations of the two clusters. **(G)** The difference in tumor grade, IDH mutation, and 1p/19q codeletion between two K-means clusters (cluster 1: *N* = 556, cluster 2: *N* = 106).

The functional enrichment analyses based on two clusters depicted distinctive pathway alterations. The ECM receptor interaction, cell adhesion molecules, other pathways were identified as key pathway associated with differentially expressed genes (DEGs) between clusters in the KEGG gene sets (Figure 2A). In REACTOME gene sets, extracellular matrix organization and interferon gamma signaling pathways were found to be over-represented in the cluster DEGs (Figure 2B).

The gene alteration analyses revealed a different gene alteration landscape between two clusters. Cluster 1 gliomas were characterized by IDH1 mutations with co-occurring TP53 and ATRX mutations or CIC, FUBP1 and NOTCH1 mutations (Figures 2C,D). Higher proportion of EGFR could be found in cluster2, which were mutually exclusive with IDH1 mutations but co-occurrent with PTEN mutations (Figures 2C,E). The analysis of CNVs demonstrated that 1p/19q co-deletion, which was recognized as an essential diagnostic marker for oligodendroglioma, mainly occurred in the cluster 1 (Figure 2F). The gain of chromosome 7 and loss of chromosome 10 (+7/-10), which was a novel diagnostic marker for glioblastoma and indicated worse prognosis, occurred more frequently in cluster 2 compared to cluster 1, in line with the worse prognosis of cluster 2 in survival analysis. Analysis of clinicopathological features of these two clusters also demonstrated that cluster 2 had higher tumor grade, lower incidence of IDH mutation, and lower incidence of 1p/19q codeletion (Figure 2G). The differences in other clinicopathological features between two clusters were given in Supplementary Figure S2.

### Analyses of immunological features in tumor microenvironment based on K-means clusters

**FIGURE 3:**
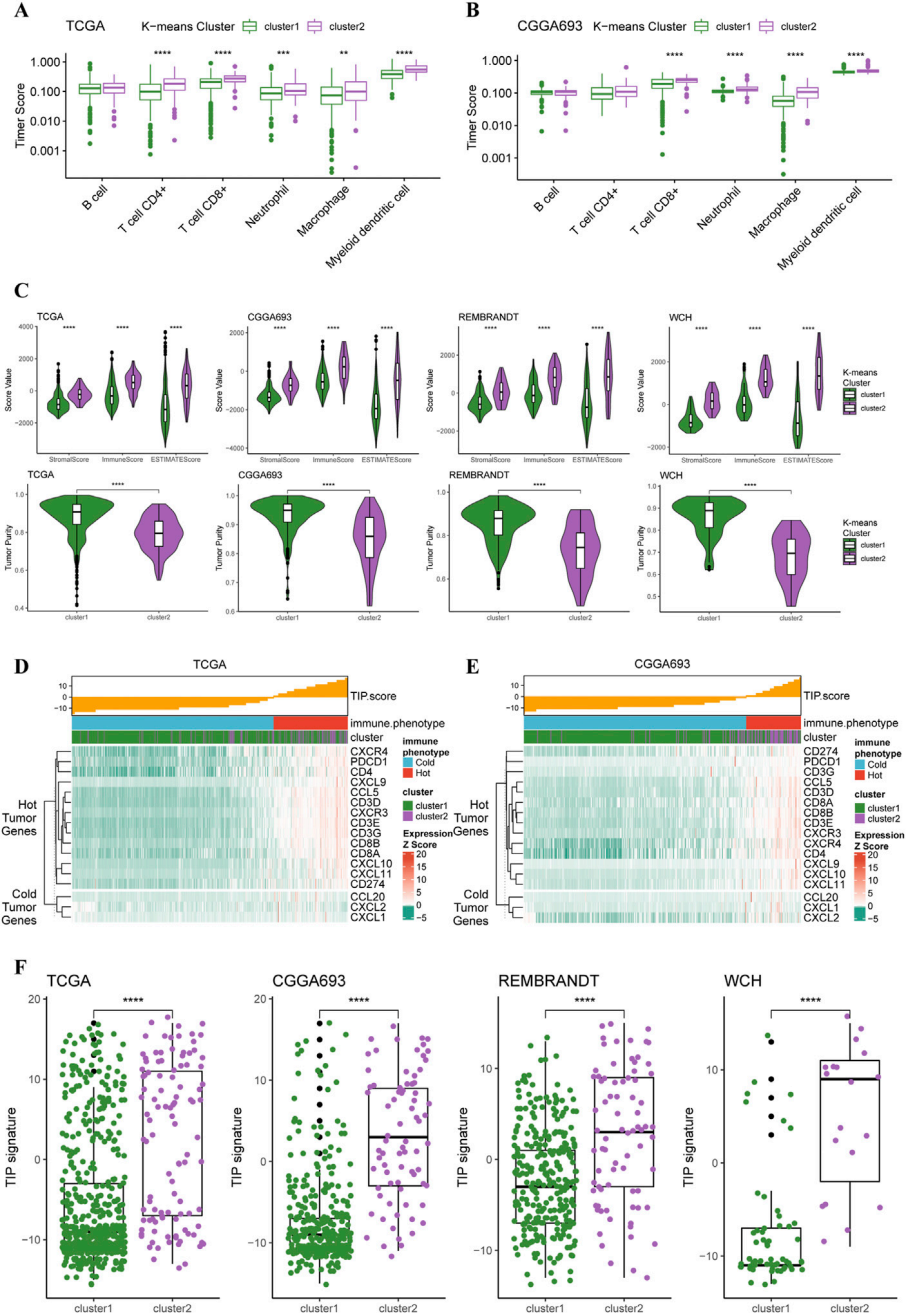
Different immunological features of tumor microenvironment between two clusters. **(A)** Boxplots of infiltration of six types of immune cells in glioma based on TIMER score in TCGA cohort (cluster 1: *N* = 556, cluster 2: *N* = 106) and **(B)** CGGA cohort (cluster 1: *N* = 339, cluster 2: *N* = 76). **(C)** Differences in stromal, immune, and ESTIMATE scores between two clusters in all four cohorts. **(D)** TIP score and related gene expression levels between two clusters in TCGA and **(E)** CGGA cohort. **(F)** Boxplots with every point on the differences in TIP score between two clusters in all four cohorts.

Based on these two K-means clusters, we conducted multiple analyses of immunological features to elucidate the differences in tumor immune landscape. The results of the TIMER score of TCGA cohort revealed that the tumor microenvironment of cluster 2 carried more macrophages, neutrophils, and CD8^+^ T-cells (Figure 3A). This conclusion could also be verified in the CGGA cohort (Figure 3B), suggesting a more complex tumor microenvironment in cluster 2. Furthermore, the results of the ESTIMATE revealed that the stromal score, immune score, and ESTIMATE score of the cluster 2 were significantly higher than cluster 1 in all four cohorts (Figure 3C). The tumor purity of cluster 2 was significantly lower than cluster 1 in all four cohorts (Figure 3C), indicating purer tumor cell microenvironment in gliomas of cluster 1. Through the calculation of TIP score, we were able to demonstrate that most gliomas of cluster 2 were more likely to be “hot” tumor immunological phenotype and expressed higher levels of gene markers for ‘hot’ tumors compared to gliomas in cluster 2 (Figure 3D). The analysis of TIP score in the CGGA cohort reached similar results (Figure 3E). Additionally, the TIP scores for the gliomas of cluster 2 were significantly higher than cluster 1 in all four cohorts (Figure 3F). All these results supported that the gliomas of cluster 2 had more complex tumor microenvironment, more immune cell infiltration, and presented with features of immunologically ‘hotter’ tumors.

### Construction and validation of the tryptophan metabolism-related genes risk signature and its relationship with clinicopathological features

**FIGURE 4:**
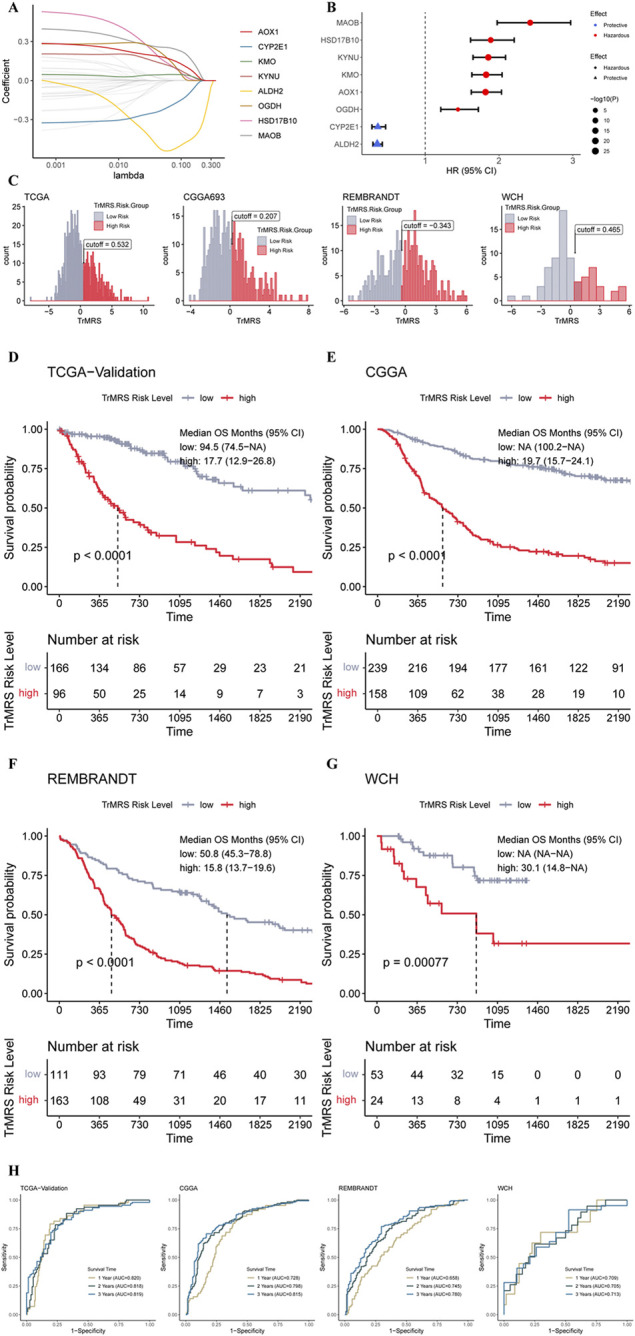
Expression signature of TrMGs and its relationship with prognosis of glioma. **(A)** Average of coefficients of eight essential TrMGs in the LASSO Cox regression at each lambda value. **(B)** The effect of each essential TrMG on the prognosis of glioma. **(C)** Histogram and optimal cut-off values of TrMRS in four cohorts (TCGA: *N* = 655 with survival data out of 662 in total, CGGA: *N* = 415, REMBRANDT: *N* = 369, WCH: *N* = 77). **(D)** K-M curve of the TCGA validation set, cut off = 0.532, **(E)** CGGA cohort, cut off = 0.207, **(F)** REMBRANDT cohort, cut off = −0.343, and **(G)** WCH cohort, cut off = 0.465. **(H)** ROC curves and matched AUC of 1-, 2-, and 3-years survival in all four cohorts.

To determine essential genes for the construction of TrMRS, we filtered the 44 TrMGs using the LASSO Cox regression in the training dataset. Subsequently, eight genes, including AOX1, CYP2E1, KMO, KYNU, ALDH2, OGDH, HSD17B10, and MAOB, were identified as essential TrMGs for the construction of TrMRS (Figure 4A). The formula to compute the TrMRS was as follows: 1.216*KYNU+0.254*KMO+0.231*AOX1+0.034*OGDH+0.006*HSD17B10 + 0.001*MAOB-0.066*ALDH2-1.064*CYP2E1.

Six genes of these essential TrMGs, including MAOB, HSD17B10, KYNU, AOX1, and OGDH, were determined as hazardous factors for glioma patients, and other two genes (CYP2E1 and ALDH2) were identified as protective factors (Figure 4B). Besides, representative immunohistochemical (IHC) staining for KYNU and ALDH2 from the Human Protein Atlas (Ponten et al., 2008) (https://www.proteinatlas.org/) was utilized to validate these results. The results of IHC demonstrated that the expression level of KYNU was higher in high-grade glioma compared to low-grade glioma (Supplementary Figure S3A), and the expression level of ALDH2 was higher in low-grade glioma (Supplementary Figure S3B), confirming the results from sequencing that KYNU was hazardous factor and ALDH2 was protective factor. Furthermore, we utilized the “surv_cutpoint” algorithm to calculate the optimal TrMRS cut-off for every cohort and allocated all the patients into TrMRS low- or high-risk groups according to this cut-off (Figure 4C). Survival analyses revealed that the overall survival of the patients in TrMRS high-risk groups was significantly poorer than TrMRS low-risk group in all four validation cohorts (Figures 4D–G), suggesting that TrMRS potentially functioned as factor for prognosis prediction. To test the efficacy of TrMRS in predicting prognosis of glioma patients, we performed ROC analyses to evaluate the performance of TrMRS alone in survival rate prediction at 1, 2, and 3 years. In the TCGA test set, the AUCs of TrMRS at 1, 2, and 3 years were 0.820, 0.818, and 0.819, respectively (Figure 4H). In other three validation cohorts, similar performances were also achieved (Figure 4H).

**FIGURE 5:**
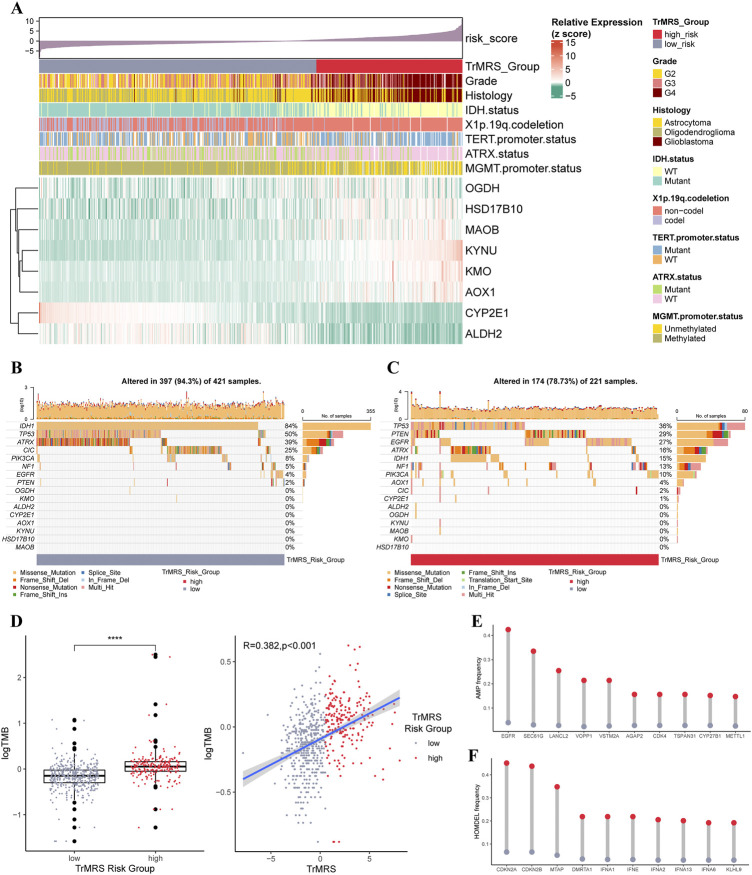
Gene mutations and copy number variations of the two risk groups. **(A)** Expression level of eight essential TrMGs and its relationship with clinicopathological features. **(B)** Gene mutations of eight essential TrMGs and top eight frequently mutated genes in TrMRS low-risk group (*N* = 429) and **(C)** high-risk group (*N* = 226). **(D)** Difference in tumor mutation burden between two TrMRS risk groups and its correlation with TrMRS. **(E)** Difference in top 10 frequent gene amplification between two risk groups. **(F)** Difference in top 10 frequent gene homozygous deletion between two risk group. *p < 0.05; **p < 0.01; ***p < 0.001; ****p < 0.0001.

The expression pattern of eight essential TrMGs was exhibited with a heatmap ordered by TrMRS (Figure 5A). The relationship of other clinicopathological features, including tumor grade, histological diagnosis, IDH mutation status, 1p/19q codeletion, TERT promoter status, ATRX status, and MGMT promoter status, with the TrMRS were also given out (Figure 5A). Further analysis of gene mutations discovered that IDH1, TP53, and ATRX were top 3 most frequently mutated genes in TrMRS low-risk group (Figure 5B). TP53, PTEN, and EGFR were top 3 most frequently mutated genes in TrMRS high-risk group (Figure 5C). Additionally, analyses of tumor mutation burden (TMB) revealed that TrMRS high-risk group harbored higher TMB than low-risk group, and the correlation analysis demonstrated that TMB was positively correlated with the TrMRS (Figure 5D). Further analysis manifested the incidence of gene amplification, including EGFR, SEC61G, and LANCL2, was significantly higher in high-risk group than low-risk group (Figure 5E). Besides, the incidence of gene homozygous deletion, including CDKN2A and CDKN2B, was also higher in high-risk group (Figure 5F).

**FIGURE 6:**
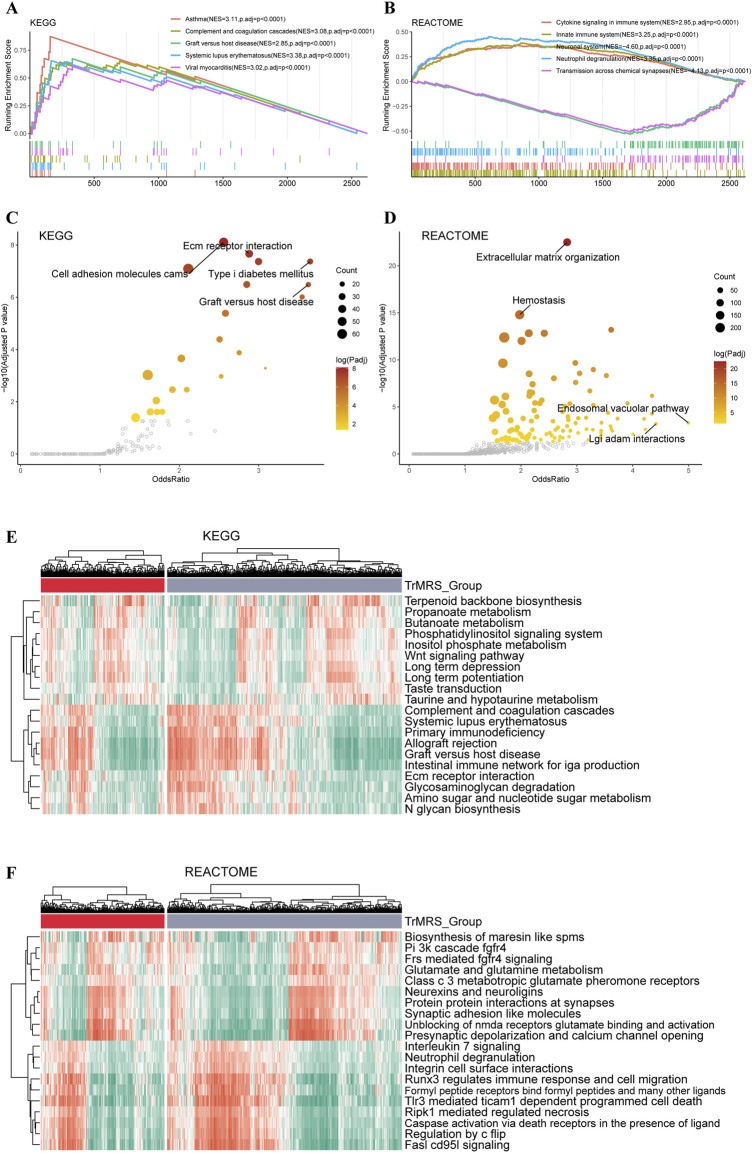
Functional enrichment analyses of the transcriptome of TrMRS risk groups. **(A)** The top five pathways with the highest normalized enrichment score in the KEGG gene sets between two risk groups. **(B)** The top five pathways with the highest normalized enrichment score in the REACTOME gene sets between two risk groups. **(C)** Pathways with high odds ratio and confidence in the KEGG and **(D)** REACTOME gene sets. **(E)** Top 20 differentially expressed KEGG gene sets (*N* = 655, low-risk group: *N* = 429, high-risk group: *N* = 226). **(F)** Top 20 differentially expressed REACTOME gene sets.

To explore the impact of TrMRS in terms of pathway, we utilized KEGG and REACTOME pathway gene sets in enrichment analyses of the DEGs between the TrMRS risk groups. The patient samples were stratified into TrMRS high- and low-risk groups. The analyses of GSEA were based on the differentially expressed genes (DEGs) between TrMRS high- and low-risk groups. The complement and coagulation cascades pathway (normalized enrichment score (NES) = 3.085, adjusted *p*-value < 0.001) and graft *versus* host disease pathway (NES = 2.855, adjusted *p*-value < 0.001) were ranked in the top five gene sets of the KEGG in the comparison between two risk groups (Figure 6A) using GSEA. Besides, the cytokine signaling in immune system pathway (NES = 2.950, adjusted *p*-value <0.001) and innate immune system pathway (NES = 3.248, adjusted *p*-value < 0.001) of REACTOME gene sets were ranked in the top five (Figure 6B). Furthermore, over-representation of the extracellular matrix receptor interaction of KEGG gene sets and the extracellular matrix organization of REACTOME gene sets were identified in the biological functions of DEGs between the two TrMRS risk groups (Figures 6C,D). Finally, the GSVA result demonstrating the top differentially expressed pathways in KEGG and REACTOME gene sets were also illustrated through heatmaps (Figures 6E,F).

### Construction of nomograms based on TrMRS and prognosis prediction

**FIGURE 7:**
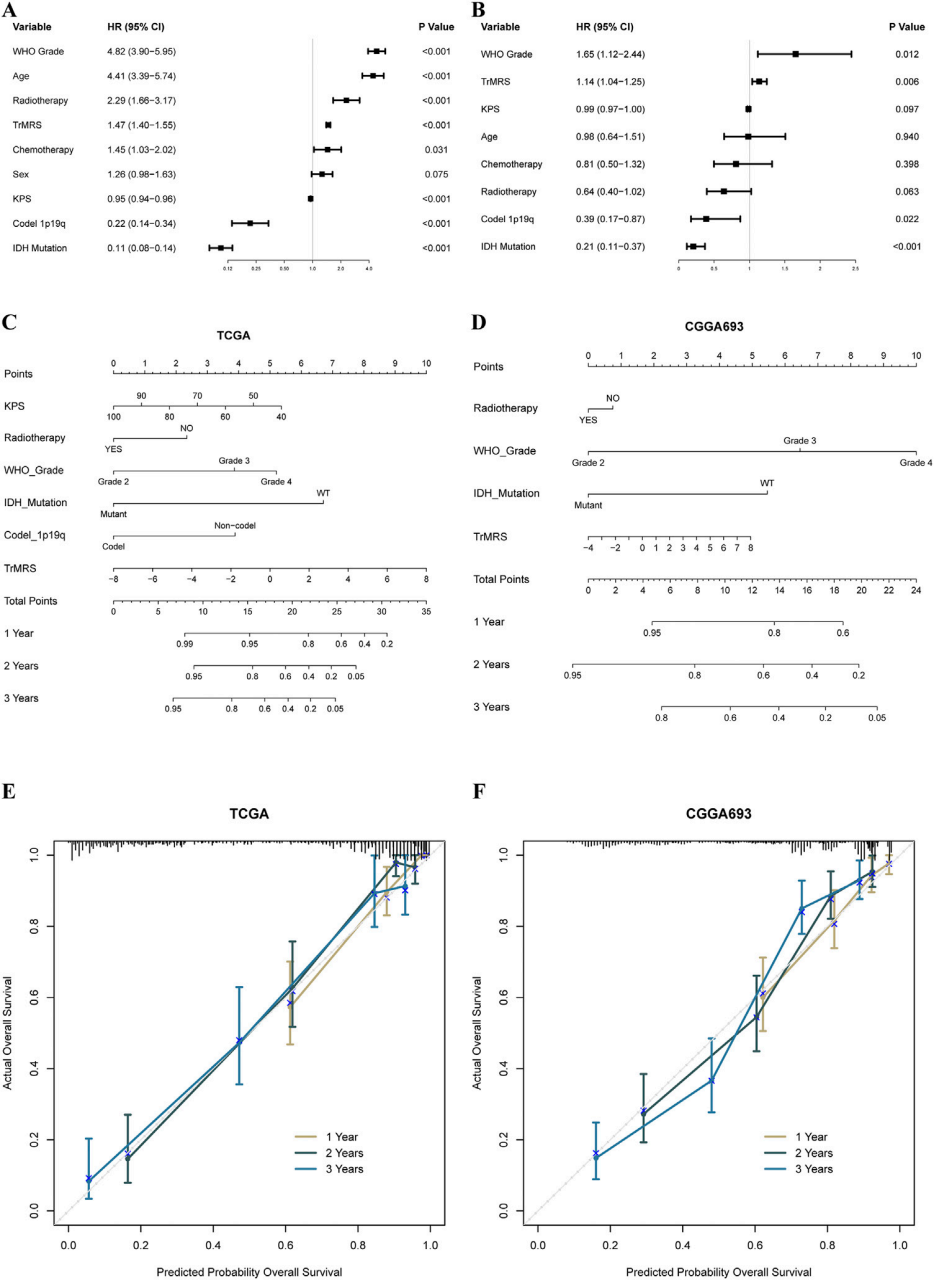
Prognostic value of TrMRS and construction of TrMRS-based nomograms. **(A)** Univariate and **(B)** Multivariate Cox regression analyses of potential prognostic factors in overall survival of gliomas. Nomogram of 1-, 2-, and 3-years survival of glioma patients based on **(C)** TCGA cohort (*N* = 655), **(D)** CGGA cohort (*N* = 415). Calibration plots of the nomogram based on **(E)** TCGA cohort and **(F)** CGGA cohort.

To construct nomograms for the prediction of glioma patients’ prognosis, we first conducted univariate followed by multivariate Cox analyses to identify potential independent prognostic factors. Result demonstrated that the tumor grade, age, radiotherapy, TrMRS, chemotherapy, 1p/19q codeletion, and IDH mutation were significant univariate prognostic factor (Figure 7A). Subsequently, these factors were enrolled in multivariate Cox regression analysis and the result revealed that TrMRS, tumor grade, 1p/19q codeletion, and IDH mutation were independent prognostic factors in glioma (Figure 7B). Eventually, these factors were combined in the construction of a nomogram for personalized survival prediction (Figure 7C). The four factors were also used to construct a nomogram for the CGGA cohort (Figure 7D). The corrected C-indexes of the nomograms based on TCGA and CGGA cohort were 0.851 and 0.779, respectively. Additionally, the 1-, 2-, and 3-years calibration curves of the nomograms also validated their efficacy in predicting survival time of glioma patients (Figures 7E,F).

### Relationship between the tryptophan metabolism-related genes and immune landscape of tumor microenvironment in glioma

**FIGURE 8:**
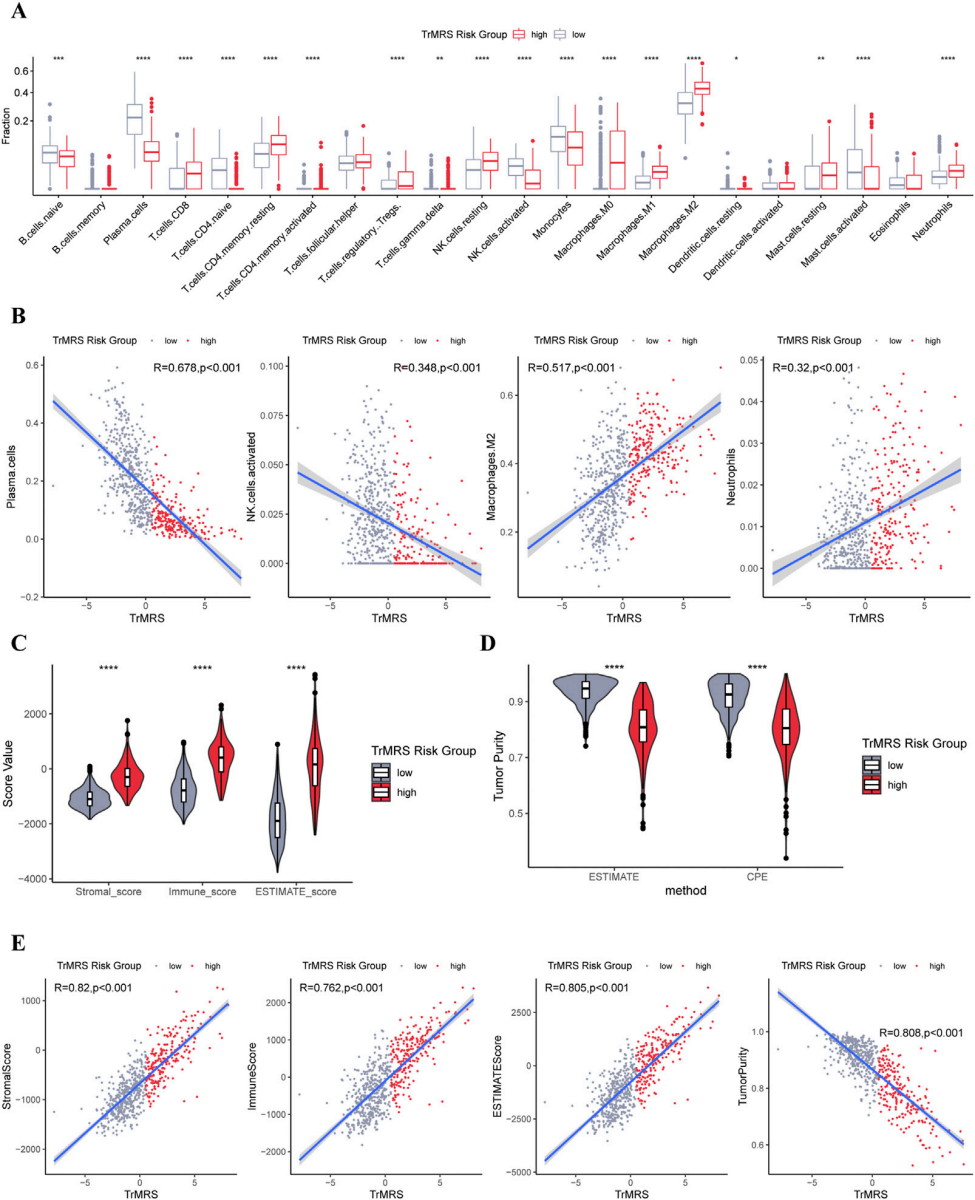
Differences in immune features of tumor microenvironment between two TrMRS risk groups. **(A)** Boxplot of the estimated fraction of 22 immune cells in tumors (low-risk group: *N* = 429, high-risk group: *N* = 226). **(B)** Analyses of correlations of TrMRS with the infiltration of plasma cells, activated NK cells, M2 macrophages, and neutrophils (*N* = 655). **(C)** Differences in the stromal, immune, and ESTIMATE scores of the two risk groups. **(D)** Tumor purity of the two risk groups based on the ESTIMATE and CPE algorithms. **(E)** Analyses of correlations of TrMRS with the stromal, immune, ESTIMATE score, and tumor purity. *p < 0.05; **p < 0.01; ***p < 0.001; ****p < 0.0001.

To elucidate the relationship between the TrMRS and tumor immune microenvironment in glioma, we conducted bundles of analyses on immunological features based on TrMRS. Firstly, the CIBERSORTx estimation of immune cell fractions depicted distinctive patterns of immune cell infiltration between two TrMRS risk groups. The TrMRS high-risk group manifested with more infiltration of resting NK cells, Macrophages (M0, M1, and M2), and neutrophils in its tumor microenvironment (Figure 8A). The low-risk group, on the other hand, had higher infiltration of activated NK cells and plasma cells. Further correlation analyses demonstrated that the infiltration of plasma cells and activated NK cells was negatively correlated with the TrMRS, and the infiltration of M2 macrophage and neutrophils was positively correlated with the TrMRS (Figure 8B). Besides, analyses of immune scores demonstrated that the TrMRS high-risk group presented with significantly higher stromal score, immune score, and ESTIMATE score (Figure 8C). The tumor purity of TrMRS high-risk group was remarkably lower than low-risk group (Figure 8D), suggesting more complicated tumor microenvironment in gliomas of TrMRS high-risk group with more immune cell infiltration. Correlation analyses revealed that the stromal score, immune score, ESTIMATE score, and tumor purity were strongly correlated with the TrMRS (R = 0.82, 0.762, 0.805, and 0.808) (Figure 8E).

**FIGURE 9:**
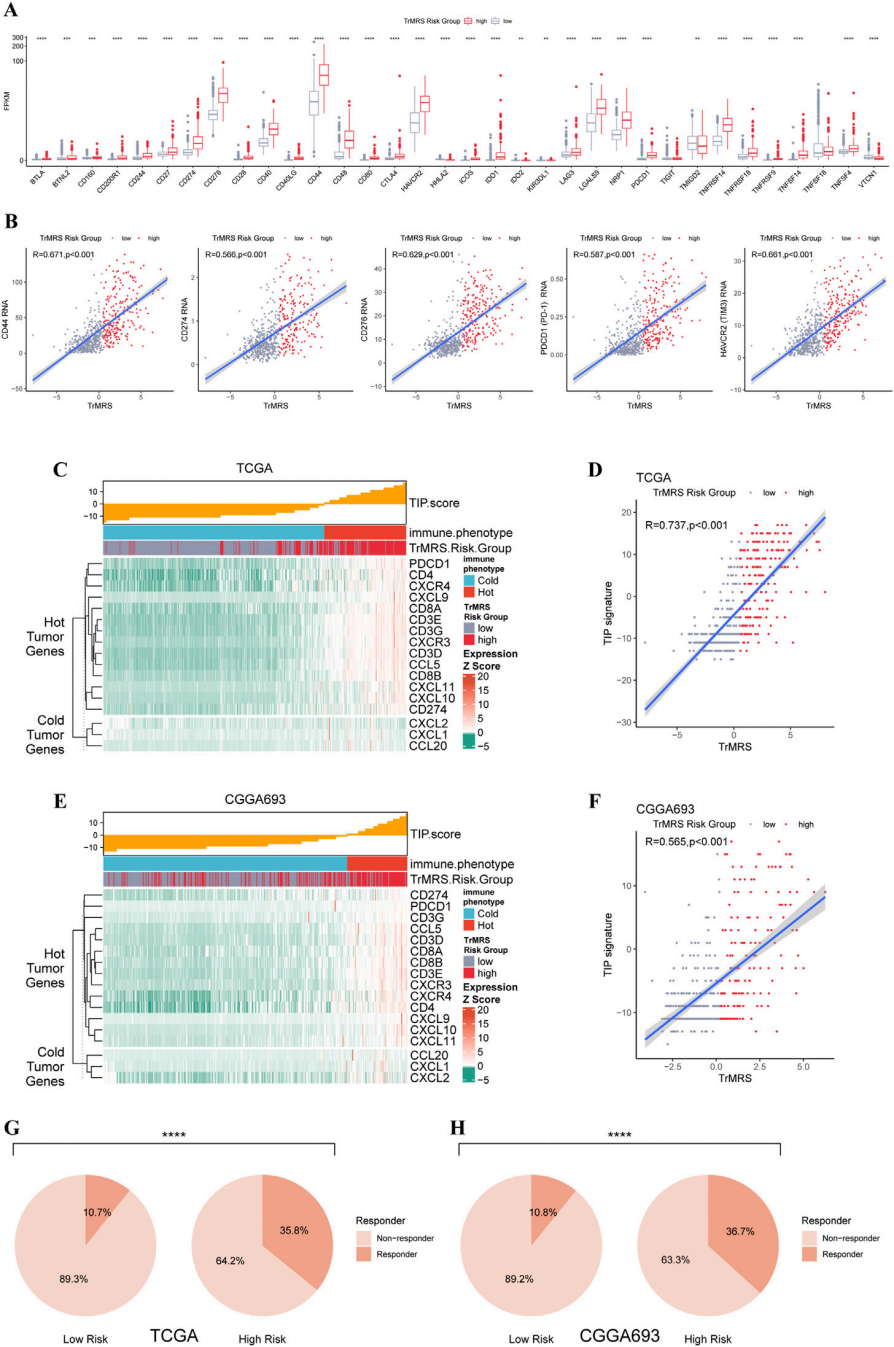
Differences in expression of immunotherapy targets and response to ICIs between two TrMRS risk groups. **(A)** The expression level of 34 immunotherapy-related genes in two risk groups (low-risk group: *N* = 429, high-risk group: *N* = 226). **(B)** Analyses of correlations of TrMRS with the expression of CD44, CD274, CD276, and PDCD1 (*N* = 655). **(C)** TIP scores and related gene expression levels between two risk groups in TCGA dataset. **(D)** Analyses of correlations of TrMRS with the TIP score in TCGA dataset. **(E)** TIP scores and related gene expression levels between two risk groups in CGGA dataset (*N* = 415, low-risk group: *N* = 249, high-risk group: *N* = 166). **(F)** Analyses of correlations of TrMRS with the TIP score in CGGA dataset. **(G)** Percentage of predicted responders to immune checkpoint inhibitors therapy in each risk group based on TCGA dataset and **(H)** CGGA dataset.

To predict potential response to immunotherapy response, we also performed analysis on the expression of immunity-related genes. Gliomas of TrMRS high-risk group presented with significantly higher expression level of CD274 (PD-L1), CD276 (B7H3), HAVCR2 (TIM3), PD1 (CD279, PDCD1), and CD44 (Figure 9A). Correlation analysis also confirmed that the expression levels of these immunotherapy-related markers were positively correlated with the value of TrMRS (Figure 9B), suggesting potential ability of TrMRS to guide the choice of immunotherapy. Furthermore, immune phenotype analysis of high- and low-risk gliomas revealed that most gliomas of TrMRS high-risk group were identified as more likely to be immunological “hot” tumors, and most tumors of low-risk group were presumed “cold” tumors (Figure 9C). A strong positive correlation between TrMRS and TIP score was also confirmed by the correlation analysis (Figure 9D). The analyses of TIP score based on the CGGA cohort also manifested with similar results (Figures 9E,F). Additionally, we utilized TIDE algorithm to predict response to immune checkpoint inhibitors. The result revealed that the glioma patients of TrMRS high-risk group were more likely to benefit from therapy of immune checkpoint inhibitors in the TCGA and CGGA cohorts (Figures 9G,H). Most above findings can be validated in the other cohorts (Supplementary Figure S4).

## Discussion

Based on the estimation in the global cancer statistics of 2020, 251 thousand death were caused by CNS malignant tumors every year (Siegel et al., 2021). Among these cases, glioma accounted for approximately 80%. Despite countless attempts worldwide to improve the clinical outcomes of glioma patients, almost no satisfactory breakthrough was achieved in recent years. For instance, the median overall survival of glioblastoma, which accounted for about 50% of all gliomas, was only 22 months after thorough treatment regime, including surgery, chemotherapy, radiotherapy, and even tumor treating field (Stupp et al., 2005; Stupp et al., 2017). Immunotherapy has proved its value in many cancers (Eggermont et al., 2018; Gandhi et al., 2018; Choueiri et al., 2021; Cortes et al., 2022). However, the application of immunotherapy in glioma faced unprecedented challenges, and all these attempts eventually failed to improve overall survival (Weller et al., 2017; Wakabayashi et al., 2018; Reardon et al., 2020; Lim et al., 2022; Omuro et al., 2022). Among all reasons, the blood-brain barrier (BBB), which could preclude most peripheral immune cells from entering central nervous system and consequently create an immunological quiescent environment, was considered important. However, an inspiring study introduced a brand-new lymphatic pathway, which permitted antigen-presenting cells to egress from brain (Louveau et al., 2015). After that, the T and B lymphocytes outside brain could be primed and then infiltrated to brain and delivered immune responses (Lim et al., 2018). These studies indicated that CNS was not a forbidden zone for immunotherapy. If we could further explore and elucidate the details of immune landscape, there were plenty of opportunities for applications of immunotherapy in gliomas.

The relationship between reshaped metabolic model of tumors and immunological landscapes has attracted surging attention (Xia et al., 2021). Several evidence have suggested that tryptophan metabolism plays a critical role in cancer (Uyttenhove et al., 2003; Muller et al., 2005). Reduced concentration has been observed in multiple cancers (Giusti et al., 1996; Huang et al., 2002), including glioma (Zhai et al., 2015). Besides, the tryptophan metabolism can regulate the T-cells and immune cell infiltration in cancer (Nakamura et al., 2007). The expression of IDO1 was also proved correlated with the immune infiltration in multiple cancers (Brandacher et al., 2006; Ino et al., 2008; Inaba et al., 2009). Therefore, to investigate whether tryptophan metabolism was correlated with malignant features and immune landscape of glioma, we analyzed the expression pattern of tryptophan metabolism-related genes in gliomas and evaluated the relationship of the TrMRS with clinicopathological features and immune landscape of gliomas.

Based on the different expression patterns of TrMGs, we allocated all patients into two K-means clusters. Subsequently, we depicted the distinctive patterns of clinicopathological features and prognosis between two clusters. The cluster 2, which presented with poorer prognosis, had a significantly higher expression level of multiple enzymes in the kynurenine pathway, including IDO1, TDO2, KYNU, and KMO, and lower expression level of KAT2, DDC, and ALDH2. IDO and TDO, which function to initiate the kynurenine pathway by converting tryptophan to N-formylkynurenine, were proved to contribute to the malignancy of glioma (Du et al., 2020), indicating that activation of kynurenine pathway was a hazardous factor for glioma, in line with our findings. Moreover, kynurenine aminotransferase 2 (KAT2), which convert kynurenine into kynurenic acid (KYNA), was manifested with higher expression level in cluster 1, suggesting protective effects of KYNA in glioma. KYNA was initially recognized with neuroprotective and anticonvulsant functions (Stone, 1993; Carpenedo et al., 2001; Erhardt et al., 2001). Besides, it has been proved that low grade gliomas synthesized more KYNA than glioblastoma (Vezzani et al., 1990). A previous study demonstrated that the concentration of KYNA was lower in the blood of glioblastoma patients compared to healthy volunteers (Adams et al., 2014). Another index for the activation of kynurenine pathway, kynurenine/tryptophan ratio, was higher in glioblastoma patients than in healthy volunteers (Adams et al., 2014). These findings were accordance with our study and suggested that activation of kynurenine pathway and inhibition of production of neuroprotective KYNA may contribute to the malignancy of gliomas. Furthermore, upregulated expression levels of DDC and ALDH, two critical enzymes in the serotonin pathway, were also confirmed in cluster 1. This phenomenon suggested that activation of serotonin pathway, which can consume tryptophan and consequently compete with kynurenine pathway for tryptophan, was related to better prognosis in glioma patients, supporting that downregulating the kynurenine pathway may reduce the malignancy of gliomas.

The incidence of gene alterations differed in these clusters. For example, IDH mutation, which has been defined as an essential marker for classification of gliomas and would result in aberrant metabolism (Yan et al., 2009; Pirozzi and Yan, 2021), mostly occurred in one of these two clusters. Because both TCA cycle and tryptophan metabolism are nicotinamide adenine dinucleotide (NAD^+^)-related pathways (Pang et al., 2021), as an essential enzyme in TCA cycle, IDH might also interact with tryptophan metabolism. Despite these potential interaction between IDH and tryptophan metabolism, the TrMRS was still identified as independent prognostic factor in multivariate analysis, which simultaneously enrolled IDH and TrMRS, suggesting that TrMRS was a strong prognostic factor in gliomas.

After monitoring TrMGs, eight TrMGs were determined as essential genes for glioma prognosis. For example, kynureninase (KYNU) is a critical enzyme in kynurenine pathway and functioned to converted kynurenine to anthranilic acid (Schwarcz and Stone, 2017). Silencing expression of KYNU could inhibit the growth of tumor cells in cutaneous squamous cells carcinoma (Ci et al., 2020). The overexpression of KYNU was also confirmed correlated with poor prognosis in gastric cancer (Zheng et al., 2020). In our study, KYNU was also confirmed as a hazardous factor in gliomas. Kynurenine monooxygenase (KMO) was a rate-limiting enzyme in kynurenine pathway and functioned to control the conversion from kynurenine to neuroactive and neurotoxic metabolites (Platten et al., 2019). Numerous studies demonstrated that KMO played a key role in tumorigenesis and tumor progression (Huang et al., 2020; Liu et al., 2021). Cytochrome P450 family two subfamily E member 1 (CYP2E1) was a critical enzyme for the metabolism of indole, which was converted from tryptophan by bacterial tryptophanases and could suppress the immune response in central nervous system (Devlin et al., 2016; Rothhammer et al., 2016). Previous study has revealed that downregulation of CYP2E1 would promote tumor progression in gliomas (Ye et al., 2021), in line with our results. Among these essential TrMGs, KYNU, and KMO are critical enzymes of tryptophan catabolism. Upregulation expression of these enzymes would subsequently increase the catabolism of tryptophan and decrease the concentration of tryptophan. KYNU and KMO were recognized as hazardous factors in previous and our studies, indicating that upregulated tryptophan catabolism and low concentration of tryptophan would lead to worse prognosis in gliomas. On the contrary, CYP2E1 was recognized as protective factor in our study, suggesting that eradication of indole, an immunosuppressive metabolites of tryptophan, would improve the prognosis of gliomas.

Further analyses of immune landscapes illustrated the relationship between tryptophan metabolism and the immune microenvironment of glioma. The CIBERSORTx analyses depicted that the infiltration of multiple immune cells, including macrophages and NK cells was correlated with the TrMRS. For example, the infiltration of M2 macrophage was positively correlated with TrMRS. M2 macrophage played a critical role in tumor promotion and immunosuppressive effects (Noy and Pollard, 2014). This result indicates that higher TrMRS value, which represents for higher tryptophan catabolism and lower level of tryptophan, is a marker for more M2 macrophage infiltration, and subsequently leads to immunosuppression. Tumors can recruit circulating monocytes and neighboring resident macrophages to their microenvironment and then polarized them from M1 to M2 macrophages, composing tumor-associated macrophages (TAMs) (Anderson et al., 2021). TAMs can produce cytokines to inhibit T-cells’ function and upregulate immunosuppressive surface proteins (Curiel et al., 2004; Colombo and Piconese, 2007; Yang and Zhang, 2017). These immunosuppressive effects of TAMs lead to immune escape in glioma and result in worse prognosis for gliomas with high TrMRS. The ESTIMATE analyses revealed that the complexity of glioma was strongly positively correlated with the TrMRS, indicating that more tryptophan catabolism and less tryptophan might help reconstruct a more complex tumor microenvironment in glioma. The expression levels of targets for ICIs, PD-1 and PD-L1, were also strongly positively correlated with the TrMRS, endorsing the potential ability of TrMRS to predict the response to ICIs. Furthermore, T-cell immunoglobulin and mucin domain-containing protein 3 (TIM3) was part of a module that contained several checkpoint receptors (Wolf et al., 2020). TIM3 frequently co-expressed with PD-1 (Sakuishi et al., 2010; Fourcade et al., 2014), which made it attractive for immunotherapy. Co-blockade of TIM3 and PD-1 can achieve greater enhancement of T-cell responses than blockade of PD-1 alone (Ngiow et al., 2011; Zhou et al., 2011). Our study demonstrated that the expression of TIM3 was strongly positively correlated with TrMRS, which also endorsed the potential ability of TrMRS to guide the application of co-blockade of TIM3 and PD-1. Hence, the correlation between TrMRS and expression of immunotherapy targets suggested that gliomas with high TrMRS would express more immunotherapy targets and have better response to immunotherapy, such as PD-1/PD-L1 inhibitors and TIM3 inhibitors. Higher TrMRS correlated with more expression of “hot tumor” features and more potential responders to ICIs. These findings demonstrated that high TrMRS would predict worse prognosis and more immunosuppressive effects. But it also predicted more expression of immunotherapy targets, which endorsed the potential ability of TrMRS to guide the application of immunotherapy.

Despite multiple analyses endorsed same results in our current study, there are still several limitations. First, protocol of sequencing and data preprocessing differed among these four independent cohorts. Second, the REMBRANDT cohort lacks some important markers, including IDH mutation status. Third, these findings of our current study still require future validation by basic experiments. Besides, due to the limitations of bulk RNA-sequencing, the potential interaction between the aberrant tryptophan metabolism in gliomas and neurons remains uncovered. However, neurons typically consist a small fraction of gliomas (Couturier et al., 2020), and is unlikely to produce significant influence on the findings of the present study. Finally, the mechanism of how tryptophan metabolism influenced immune landscape of gliomas remains unclear and requires further exploration.

## Conclusion

In conclusion, we revealed that the expression pattern of TrMGs was closely correlated with clinicopathological and immunological features in glioma. The novel tryptophan metabolism evaluation score system, TrMRS, showed for strong ability to predict prognosis of glioma patients. Moreover, higher TrMRS, representing for more active tryptophan catabolism and less tryptophan, predicts more immune infiltration, immunosuppression and, more targets for immunotherapy, endorsing the usages of TrMRS in guiding immunotherapy in gliomas.

## Data Availability

The datasets presented in this study can be found in online repositories. The names of the repository/repositories and accession number(s) can be found in the article/Supplementary Material.

## Supplementary material

SUPPLEMENTARY FIGURE S1
**(A)**Average silhouette width corresponding to each cluster number (k) in K-means clustering of the gliomas based on TrMG expression. **(B)** Heatmap of expression of 44 TrMGs based on two clusters. **(C)** Differences in the expression of 44 TrMGs between two K-means clusters. **p* < 0.05; ***p* < 0.01; ****p* < 0.001; *****p* < 0.0001.

SUPPLEMENTARY FIGURE S2 Differences in the clinicopathological features between two K-means clusters. **p* < 0.05; ***p* < 0.01; ****p* < 0.001; *****p* < 0.0001.

SUPPLEMENTARY FIGURE S3 Representative immunohistochemical staining for KYNU and ALDH2 from the Human Protein Atlas. **(A)**Representative staining for KYNU in high- and low-grade glioma (https://www.proteinatlas.org/ENSG00000115919-KYNU/, antibody: HPA031686). **(B)** Representative staining for ALDH2 in high- and low-grade glioma (https://www.proteinatlas.org/ENSG00000111275-ALDH2/, antibody: HPA051065).

SUPPLEMENTARY FIGURE S4 Differences in immunological characteristics of tumor microenvironment between two TrMRS risk groups in CGGA, REMBRANDT, and WCH cohorts. **p* < 0.05; ***p* < 0.01; ****p* < 0.001; *****p* < 0.0001.
