# Supplementary figures and images for: Prognosis prediction and tumor immune microenvironment characterization based on tryptophan metabolism-related genes signature in brain glioma
Source: Front Pharmacol. 2022 Nov 1;13:1061597. doi: 10.3389/fphar.2022.1061597 (PMC9663932; doi:10.3389/fphar.2022.1061597)

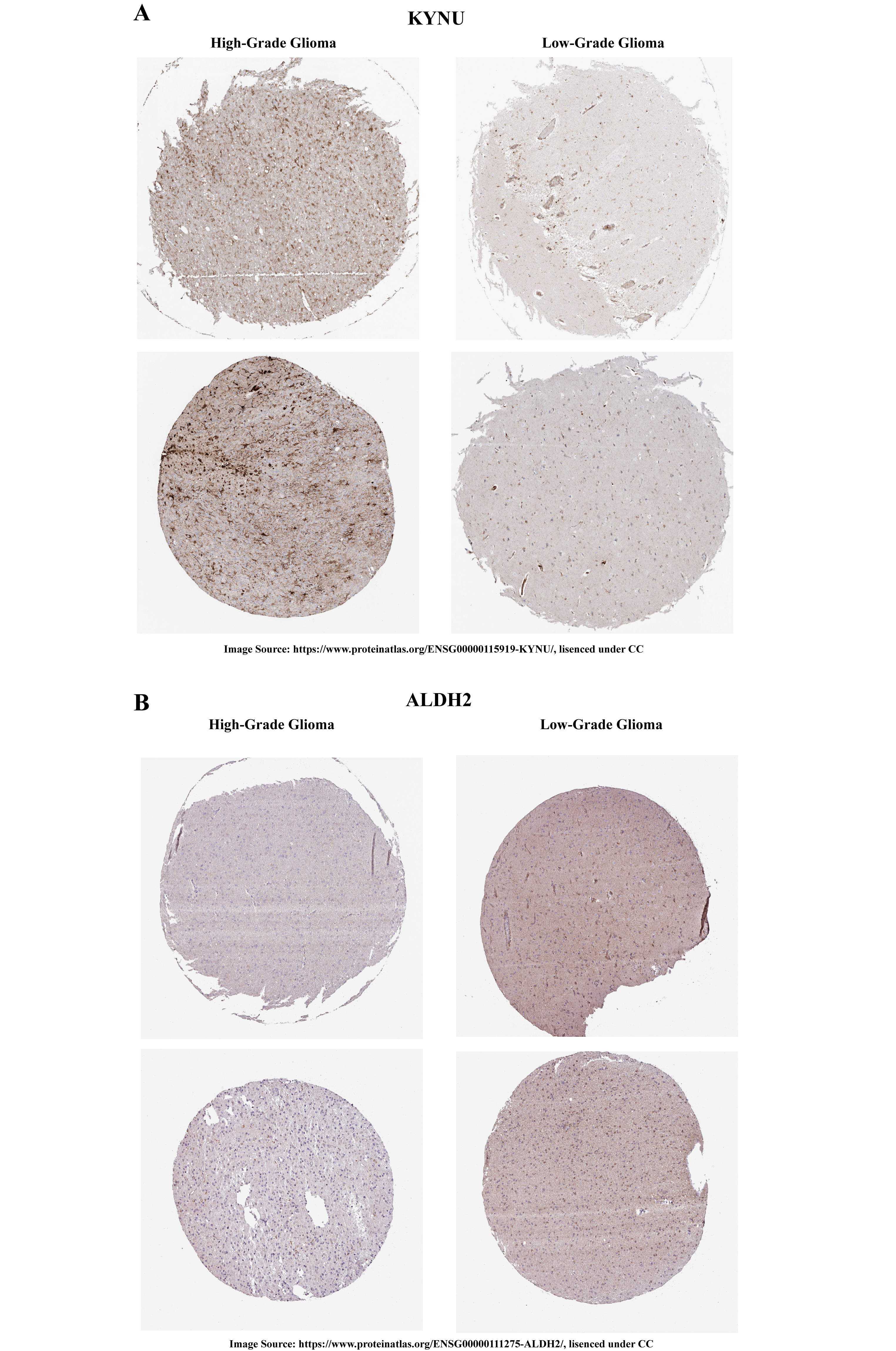

Supplement: Supplementary file 1 [file Image3.TIF]

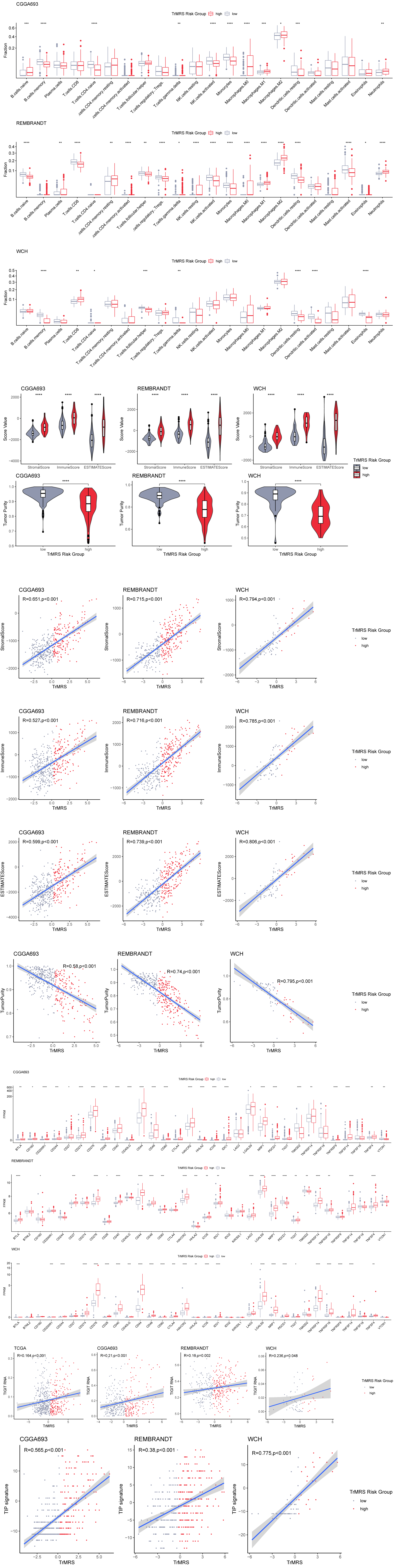

Supplement: Supplementary file 2 [file Image4.TIF]

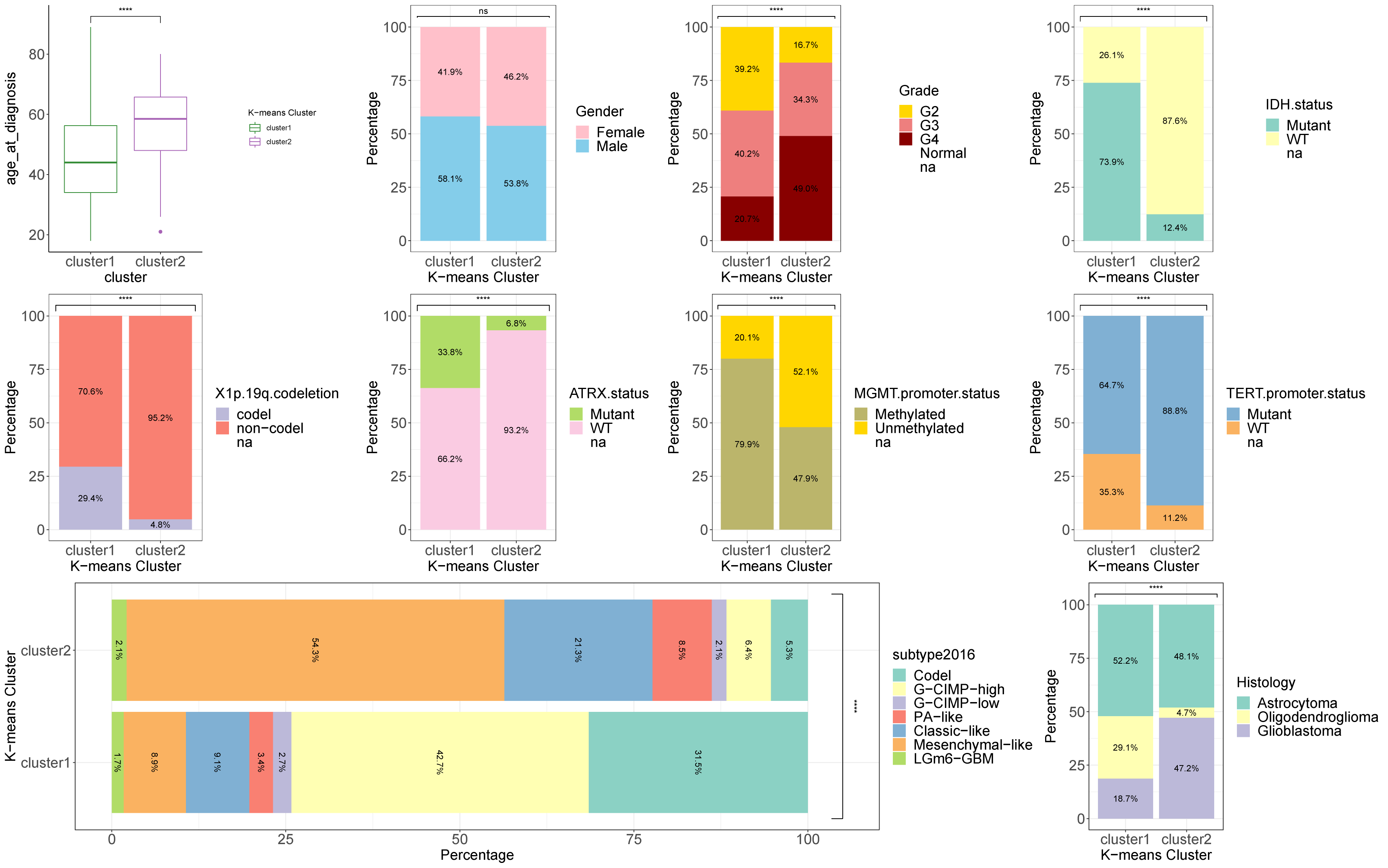

Supplement: Supplementary file 3 [file Image2.TIF]

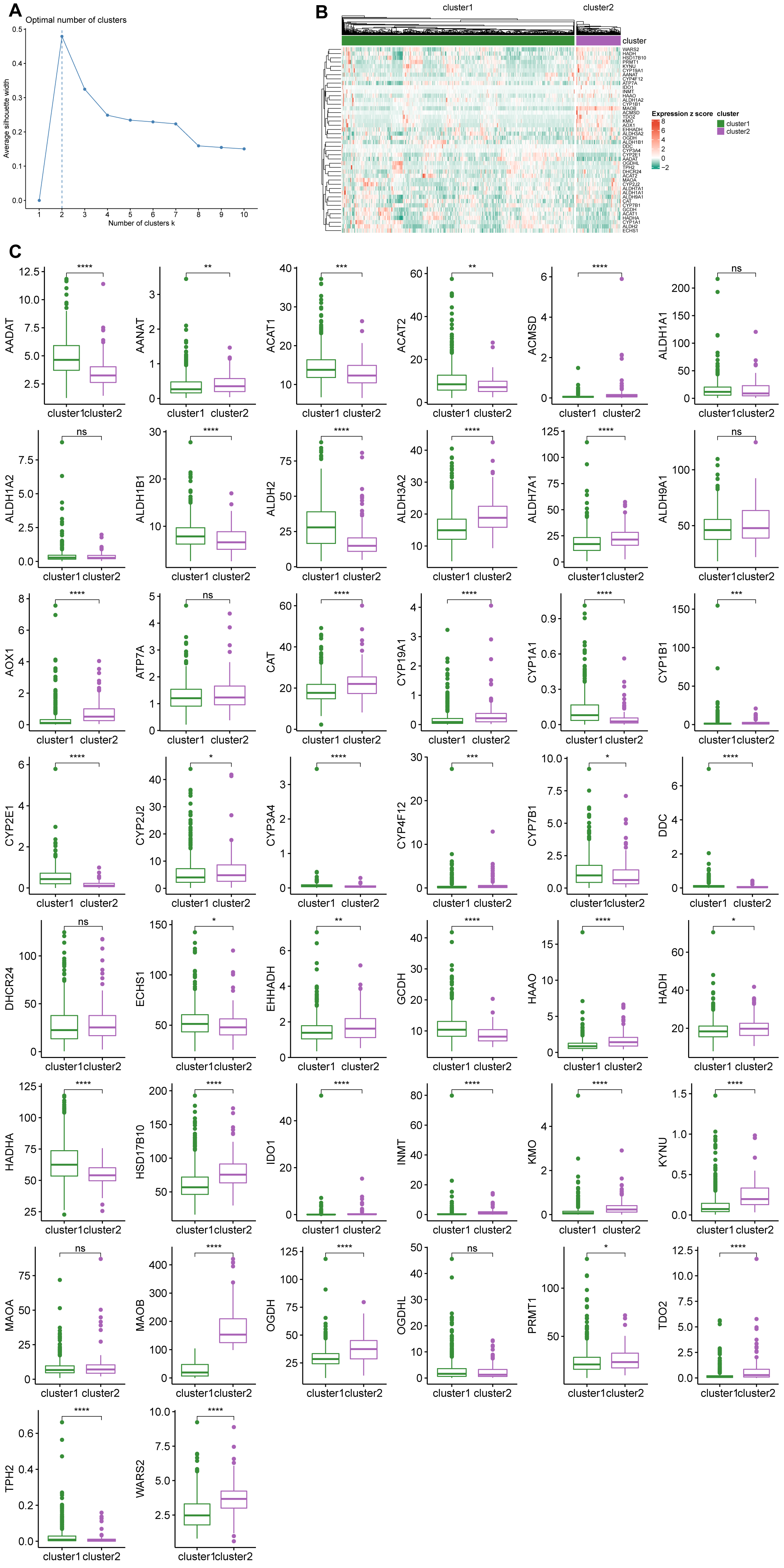

Supplement: Supplementary file 4 [file Image1.TIF]
